# Development of Kamala-Based, a Thai Traditional Remedy, Nanoemulsion Gel and In Vitro Release Behavior of Phenylbutenoid Markers

**DOI:** 10.3390/gels12050415

**Published:** 2026-05-09

**Authors:** Siraporn Mahakoat, Sujaree Panomket, Catheleeya Mekjaruskul, Bunleu Sungthong

**Affiliations:** 1 Master of Science Program in Herbals and Thai Traditional Medicine, Faculty of Pharmacy, Mahasarakham University, Maha Sarakham 44150, Thailand; 66010781003@msu.ac.th; 2 Kamalasai Hospital, Kalasin 46130, Thailand; panomketsujaree@gmail.com; 3 Integrative Pharmaceuticals and Innovation of Pharmaceutical Technology Research Unit, Faculty of Pharmacy, Mahasarakham University, Maha Sarakham 44150, Thailand; catheleeya.m@msu.ac.th

**Keywords:** Kamala extract, knee osteoarthritis, nanoemulsion gel, phenylbutenoids, ultrasonication

## Abstract

Kamala is a traditional Thai herbal knee poultice containing phenylbutenoid compounds with potent anti-inflammatory activity; however, its conventional form is inconvenient to use and exhibits variability in active compound content. This study aimed to develop a Kamala-based nanoemulsion gel to enhance dermal delivery and improve formulation consistency. Oils, surfactants, and co-surfactants were screened for their solubilization efficiency of (*E*)-1-(3,4-dimethoxyphenyl)butadiene (DMPBD) and (*E*)-4-(3′,4′-dimethoxyphenyl)but-3-en-1-ol (Compound D) using GC–MS. Pseudo-ternary phase diagrams were constructed to identify isotropic regions, and nanoemulsions with different Smix ratios were prepared by ultrasonication. Droplet size, polydispersity index (PDI), and short-term stability were evaluated. The optimized nanoemulsion was incorporated into a gel, and in vitro release was assessed using Franz diffusion cells. Coconut oil exhibited the highest solubilization capacity for both markers. A Tween 80:n-butanol system (2:1) generated the largest isotropic region (22.88%). The optimized formulation (Kamala extract:coconut oil:Smix:water = 1:2:50:47) showed droplet sizes of 77.92 ± 8.34 nm at 0 h and 130.89 ± 29.16 nm at 72 h, with PDI < 0.20. The nanoemulsion gel prepared with Aristoflex Velvet^®^ (1% *w*/*w*) was transparent and physically stable. Franz diffusion studies demonstrated enhanced cumulative release and flux of Compound D in PBS containing 1% Tween 80. These findings indicate that the Kamala nanoemulsion gel is a promising topical delivery system for phenylbutenoid compounds in knee osteoarthritis.

## 1. Introduction

Knee osteoarthritis (OA) is a chronic degenerative joint disease characterized by progressive cartilage degradation, inflammation, pain, and functional impairment, predominantly affecting older adults and leading to substantial reductions in mobility and quality of life [1]. The global prevalence of knee OA continues to increase with aging populations, imposing a significant socioeconomic burden. Although conventional management strategies—including nonsteroidal anti-inflammatory drugs (NSAIDs), intra-articular corticosteroid injections, and physical rehabilitation—can provide symptomatic relief, their long-term use is frequently limited by adverse effects and suboptimal patient adherence [2,3]. Consequently, there is a growing demand for safer, effective, and patient-friendly topical therapies suitable for chronic use [2,3].

Natural products and traditional herbal formulations have gained renewed attention as alternative or complementary approaches for OA management due to their multitarget pharmacological actions and generally favorable safety profiles [4]. In Thai traditional medicine, the Kamala knee poultice (Thai: Yapok-Kamala) has been used for centuries to alleviate knee pain and inflammation [4]. This traditional formulation consists of a synergistic combination of medicinal plants (1300 g), containing 500 g *Zingiber montanum* rhizome, and 100 g of each *Materia Medica*; *Zingiber officinale* rhizome, *Curcuma zedoaria* rhizome, *Piper retrofractum* fruit, *Piper sarmentosum* root, *Nigella sativa* seed, *Nigella damascene* seed, *Crinum asiaticum* leaf, and *Dracaena angustifolia* leaf. Among its phytochemical constituents, phenylbutenoids—particularly (*E*)-1-(3,4-dimethoxyphenyl)butadiene (DMPBD) and (*E*)-4-(3′,4′-dimethoxyphenyl)but-3-en-1-ol (Compound D)—have been reported to exhibit potent anti-inflammatory and chondroprotective activities, highlighting their suitability as chemical markers for formulation standardization [4]. However, the conventional application of Kamala as a thick herbal paste requiring prolonged application is inconvenient, poorly reproducible, and limits widespread clinical adoption.

Advances in nanotechnology offer promising strategies for modernizing traditional herbal formulations [5,6]. Nanoemulsions, defined as kinetically stable oil-in-water systems with droplet sizes typically below 200 nm, provide several advantages, including enhanced solubilization of lipophilic compounds, improved dermal penetration, high interfacial surface area, optical clarity, and improved physical stability [5,7]. When incorporated into a gel matrix, nanoemulsion gels combine the penetration-enhancing properties of nanoemulsions with the practical benefits of gels, such as ease of application, prolonged residence time on the skin, and improved sensory acceptance [7,8]. These attributes are particularly important for chronic conditions such as knee OA, where sustained topical treatment and long-term patient adherence are essential.

Previous studies have demonstrated the potential of nanoemulsion-based systems to enhance the transdermal delivery of herbal extracts and poorly water-soluble phytochemicals [7,9]. Nevertheless, to date, the systematic development of a nanoemulsion gel derived from the traditional Kamala poultice has not been reported. Critical formulation aspects—including the solubilization behavior of phenylbutenoid markers, optimization of surfactant and co-surfactant systems, phase behavior characterization, and physicochemical stability—remain insufficiently explored, representing major barriers to the translation of the traditional Kamala formulation into a standardized and clinically applicable topical nanopharmaceutical [10].

Recent studies have highlighted the potential of nanoemulsion-based and nanoemulgel systems in enhancing topical drug delivery for osteoarthritis and inflammatory skin applications by improving drug solubility, skin permeability, and sustained release behavior [11,12,13,14].

To address these challenges, the present study aimed to develop and optimize a Kamala-based nanoemulsion gel using a rational formulation approach integrating pseudo-ternary phase diagram construction and high-energy ultrasonication [5,6]. Oils, surfactants, and co-surfactants were systematically screened to maximize the solubilization of DMPBD and Compound D. The optimized nanoemulsion was subsequently incorporated into gel matrices and evaluated for physicochemical properties, stability, and droplet characteristics. Furthermore, in vitro release and diffusion studies using Franz diffusion cells were conducted to elucidate the dermal release behavior of the phenylbutenoid markers and to identify suitable receptor media for maintaining sink conditions [9,15].

This work represents the first comprehensive investigation into the development of a nanoemulsion gel based on the traditional Kamala formulation. By establishing standardized formulation parameters and demonstrating enhanced release performance of key bioactive markers, this study provides a scientifically validated and modernized platform for advancing Kamala-based topical products toward further preclinical and clinical evaluation.

## 2. Results and Discussion

The results of this study demonstrate a systematic and rational formulation strategy for modernizing the traditional Kamala knee poultice into a nanoemulsion gel suitable for topical application. The findings are discussed in the context of formulation science, dermal drug delivery principles, and previous reports on nanoemulsion-based systems, with emphasis on their implications for knee osteoarthritis management [5,7,9].

The enhanced release and diffusion behavior observed in this study is consistent with previous reports demonstrating that nanoemulsion-based systems can improve the bioavailability of lipophilic compounds by increasing their solubilization and facilitating dermal transport [16,17,18]. The incorporation of nanoemulsions into gel matrices further enhances formulation stability and prolongs residence time on the skin, supporting sustained drug delivery.

### 2.1. Screening of Oils for Solubilization of Phenylbutenoid Markers

**Figure 1:**
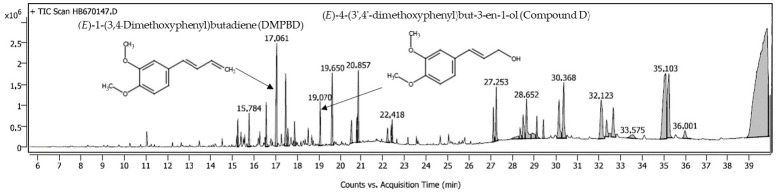
GC-MS Chromatogram of phenylbutenoids in Kamala coconut oil.

The solubility of bioactive compounds in the oil phase is a fundamental factor governing nanoemulsion formation, stability, and subsequent dermal delivery performance [5,7]. In this study, five oils—sesame, palm, rice bran, coconut, and olive oils—were evaluated for their capacity to solubilize the phenylbutenoid markers using GC–MS analysis, as shown in Figure 1. Identifications of the bioactive markers were assessed using retention times of DMPBD (approximately 17 min) and compound D (approximately 19 min) and their corresponding mass fragmentation patterns of the NIST 17 mass spectral library. Among the tested oils, coconut oil exhibited markedly superior solubilization efficiency for both markers, as evidenced by significantly higher chromatographic peak areas compared with the other oils (Table 1).

High solubilization capacity within the oil phase is essential to prevent drug precipitation during emulsification and storage, which can otherwise lead to phase instability and reduced bioavailability [5,10]. The favorable performance of coconut oil is consistent with previous reports indicating that coconut oil saturated fatty acids approximately 92% with primarily lauric acid (C12:0) at 47.70% and myristic acid (C14:0) at 19.90% [11]. Its high content of medium-chain triglycerides facilitates the solubilization of lipophilic phenylbutenoid structures and enhances their partitioning into stratum corneum lipids, thereby supporting dermal penetration [7,9]. These characteristics justified the selection of coconut oil as the primary oil phase for further nanoemulsion development.

### 2.2. Screening of Surfactant and Co-Surfactant Systems

Surfactants play an essential role in the formation of conventional emulsions and nanoemulsions by reducing the interfacial tension between oil and aqueous phases, thereby improving miscibility and system stability. For oil-in-water (O/W) nanoemulsion systems, surfactants with relatively high hydrophilic–lipophilic balance (HLB) values are generally preferred to facilitate efficient dispersion of the oil phase in water. In combination with co-surfactants, these systems further enhance interfacial flexibility and promote droplet size reduction during ultrasonication [5,11].

Three surfactants were selected for preliminary screening to identify the most suitable candidate for development of the Kamala nanoemulsion gel: Tween 80^®^ (Polysorbate 80), Span 80^®^ (Sorbitan monooleate), and Cremophor RH40^®^ (PEG-40 Hydrogenated Castor Oil). The screening procedure was adapted from a previously reported method [12]. Briefly, 15% (*w*/*w*) aqueous surfactant solutions were prepared, and coconut oil containing Kamala extract (KCO) was added incrementally at 2 µL per addition under vigorous vortex mixing. The clarity of the system was visually observed after each addition. If the solution remained clear, additional oil was added until turbidity appeared. The maximum amount of KCO that could be incorporated while maintaining a clear one-phase system was recorded and used to compare the solubilization efficiency of each surfactant (Table 2).

Tween 80 and Cremophor RH40 exhibited the highest oil incorporation capacity, whereas Span 80 showed lower tolerance. However, Tween 80 produced the clearest and most physically stable system, while Cremophor RH40 resulted in a slightly turbid dispersion. Therefore, Tween 80 was selected as the optimal surfactant for subsequent nanoemulsion development.

**Figure 2:**
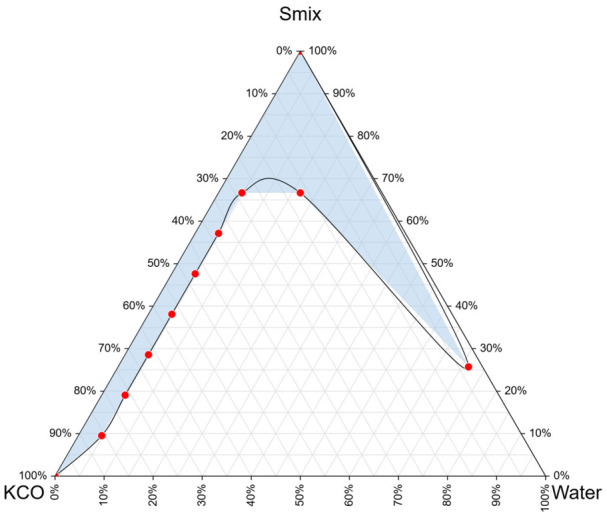
Pseudo-ternary phase diagram showing one-phase regions for Smix, KCO, and water (light blue region). Red dot is an experimental combination of Smix, KCO, and water in different ratios. Smix is a combination of Tween 80 and n-butanol. KCO is a coconut oil containing Kamala extract.

For co-surfactant screening, four candidates, namely absolute ethanol, propylene glycol, isopropyl alcohol, and n-butanol, were further evaluated by constructing pseudo-ternary phase diagrams. The percentages of one-phase isotropic nanoemulsion regions were 11.24%, 14.63%, 16.51%, and 22.88%, respectively. Among these candidates, n-butanol produced the broadest nanoemulsion region as shown in Figure 2, indicating superior interfacial film flexibility and enhanced self-emulsification capacity. Therefore, n-butanol was selected as the optimal co-surfactant for further formulation development [5,13].

### 2.3. Optimization of Smix Ratio Using Pseudo-Ternary Phase Diagrams

Three surfactants were screened to identify the most suitable candidate for nanoemulsion development based on coconut oil incorporation capacity, visual appearance, and physical stability (Table 2). Tween 80 and Cremophor RH40 showed the highest oil incorporation capacity, each tolerating 4 µL of coconut oil without phase separation, whereas Span 80 tolerated only 2 µL (*p* < 0.05).

Although Tween 80 and Cremophor RH40 showed comparable incorporation capacity, Tween 80 produced a clear and stable system, while Cremophor RH40 yielded a slightly turbid dispersion. In contrast, Span 80 generated a turbid and unstable system. Based on the combined criteria of solubilization efficiency, clarity, and physical stability, Tween 80 was selected as the optimal surfactant for subsequent nanoemulsion formulation (Table 2).

Following surfactant selection, Tween 80 was combined with n-butanol at different Smix ratios and evaluated using pseudo-ternary phase diagrams. The Smix ratio of 2:1 (Tween 80:n-butanol) generated the broadest one-phase nanoemulsion region, indicating superior self-emulsification efficiency and system stability. In contrast, formulations containing excessive co-surfactant showed reduced stability, likely due to over-penetration of n-butanol into the interfacial film, which weakened interfacial cohesion. Conversely, insufficient co-surfactant content may have resulted in rigid interfacial layers that restricted droplet curvature and limited nanoemulsion formation [5,10].

The 2:1 Smix ratio therefore represents an optimal balance between interfacial tension reduction and steric stabilization. This finding is consistent with Derjaguin–Landau–Verwey–Overbeek (DLVO) theory and previous studies reporting improved nanoemulsion stability at intermediate surfactant–co-surfactant ratios [5,14].

### 2.4. Nanoemulsion Formulation Development

Nanoemulsions containing different Smix concentrations were prepared using ultrasonication, a high-energy method that promotes droplet breakup through acoustic cavitation [16].

The physicochemical characteristics of nanoemulsion formulations containing different Smix concentrations are summarized in Table 3. Immediately after ultrasonication, K1 and K2 showed significantly smaller particle sizes than K3 (*p* < 0.05), while no significant difference was observed between K1 and K2. After 72 h of storage, K2 maintained the smallest droplet size (130.89 ± 29.16 nm) and the lowest PDI (0.195 ± 0.013), indicating superior short-term stability and a narrow particle size distribution.

K1 showed a significant increase in particle size and PDI after 72 h (*p* = 0.010 and 0.004, respectively), suggesting droplet aggregation or instability during storage. K2 showed a significant increase in particle size (*p* = 0.039) but no significant change in PDI (*p* = 0.885), indicating that although slight droplet growth occurred, the system remained relatively homogeneous. In contrast, K3 showed no statistically significant changes in either particle size or PDI (*p* > 0.05), but exhibited larger droplet size overall [17].

These findings indicate that the formulation containing 50% Smix (K2) provided the most favorable balance between droplet size reduction and colloidal stability.

**Figure 3:**
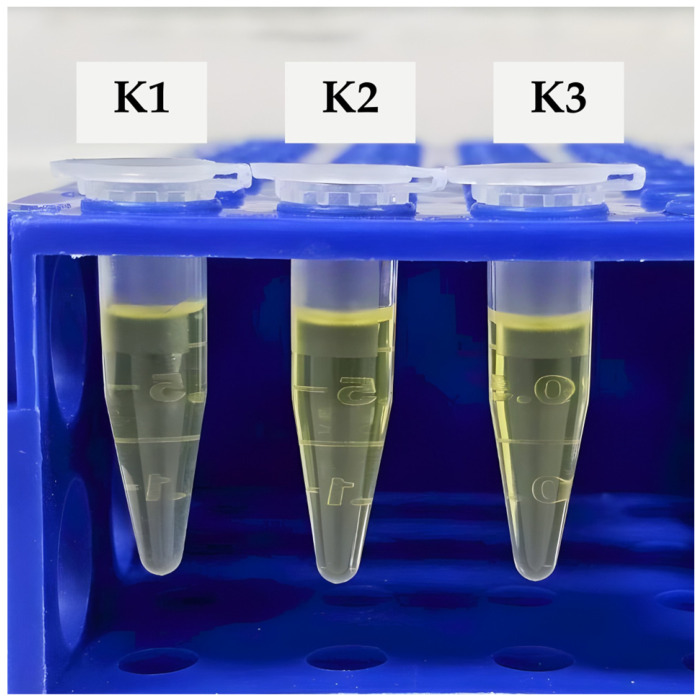
Representative images showing the physical appearance of short-term stability of nanoemulsion formulations K1–K3.

Although a modest increase in droplet size was observed during storage, the absence of phase separation and the maintenance of low polydispersity indicate good kinetic stability and minimal Ostwald ripening [5,14]. Excessive Smix content, as observed in formulation K3, increased system viscosity and hindered efficient droplet disruption, underscoring the importance of compositional balance rather than maximal surfactant loading [10]. Based on these considerations, formulation K2 was selected as the optimized nanoemulsion system (Figure 3).

### 2.5. Transition to Nanoemulsion Gel: Selection of Gelling Agents

To improve practicality and patient acceptability, the optimized nanoemulsion was incorporated into various gel matrices. Among the evaluated gelling agents, Aristoflex Velvet at 1% *w*/*w* produced a transparent, homogeneous nanoemulsion gel with stable viscosity across different storage temperatures (Table 4).

This polymer forms an elastic three-dimensional network that is compatible with high surfactant concentrations and maintains clarity, making it particularly suitable for nanoemulsion-based topical formulations [8,18].

The resulting gel exhibited skin-compatible pH values (5.25 ± 0.02 to 6.05 ± 0.01) within the physiological acid mantle range, supporting its suitability for repeated dermal application [10]. These rheological and physicochemical properties are especially relevant for knee osteoarthritis management, where ease of spreading, residence time on the skin, and user comfort are critical for long-term adherence.

The physical stability of the nanoemulsion gel was assessed over a 60-day period under three storage conditions (5 °C, 25 °C, and 40 °C), following established cosmetic stability testing guidance (ISO/TR 18811:2018) and aligned with the general principles of accelerated stability assessment described in the ASEAN Guideline on Stability Study of Drug Product (R1) [19,20].

**Figure 4:**
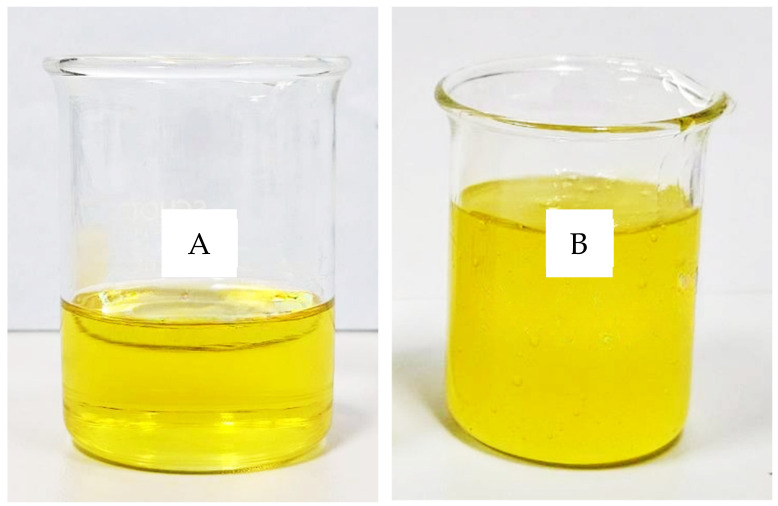
Representative images of the optimized K2 nanoemulsion before gel formation (**A**) and the K2 nanoemulsion gel after incorporation of 1% Aristoflex Velvet (**B**), showing successful sol-to-gel transition.

Throughout the study period, the formulation maintained physical stability under all storage conditions, with no evidence of phase separation, creaming, or sedimentation. The gel retained its characteristic transparency and homogeneity, indicating the absence of visible macroscopic destabilization (Figure 4).

A simplified comparison of viscosity stability at 25 °C over 60 days is presented in Table 4. Among the tested gel-forming agents, Aristoflex Velvet 1% exhibited the smallest viscosity change (−3.26%) with no statistically significant difference from baseline (*p* = 0.421), indicating excellent rheological stability. In contrast, Aristoflex AVC 1% showed a significant increase in viscosity (+23.59%, *p* < 0.001), suggesting progressive thickening during storage. Sepimax ZEN 0.5% demonstrated a significant viscosity reduction (−17.15%, *p* = 0.002), indicating insufficient gel network integrity. Although Sepimax ZEN 1% remained relatively stable (+7.16%, *p* = 0.084), its initial viscosity was comparatively high, which may adversely affect spreadability and ease of application.

Based on these findings, Aristoflex Velvet 1% was selected for further formulation development. The optimized nanoemulsion gel showed no significant viscosity change after 60 days at 25 °C (*p* = 0.421), confirming good rheological stability under ambient storage. Although statistically significant increases were observed at 5 °C and 40 °C, the magnitude of change remained moderate (+12.30% and +4.26%, respectively), and no visible phase separation or syneresis was detected. These results support the suitability of Aristoflex Velvet 1% as an effective gel-forming agent for the developed nanoemulsion system (Table 4).

The physicochemical stability of the system is ascribed to the rational formulation design. Specifically, the selection of Tween 80 and n-butanol as surfactant and co-surfactant, respectively, at an optimized Smix ratio, ensured adequate interfacial film stabilization and suppressed droplet coalescence. The integration of Aristoflex Velvet as a polymeric gelling agent further reinforced the structural integrity of the formulation by restricting internal phase mobility and minimizing the thermodynamic driving force for phase separation.

Although colloidal parameters including droplet size and zeta potential were not monitored longitudinally during storage, the low polydispersity index (PDI < 0.3) recorded at the time of preparation, together with the sustained macroscopic homogeneity, provides indirect evidence of kinetic stability throughout the observation period. These findings are in agreement with the literature, which reports that gel-embedded nanoemulsion systems exhibit enhanced physical stability as a result of reduced droplet diffusivity and the mechanical support provided by the polymer network [12,13].

Certain limitations of the present stability assessment merit acknowledgment. Comprehensive long-term stability data under real-time and accelerated conditions over a 3–6 month period were not obtained, and freeze–thaw cycling was restricted to three consecutive cycles. These constraints may limit the extrapolation of the current findings to long-term storage scenarios and should be addressed in subsequent investigations.

Collectively, the results demonstrate that the nanoemulsion gel possesses satisfactory short- to medium-term physical stability, lending support to its potential application as a stable platform for topical delivery.

### 2.6. In Vitro Release Study via Franz Diffusion Cells

#### 2.6.1. Release of DMPBD

DMPBD exhibited minimal release in both receptor media throughout the experimental period, with concentrations remaining below the limit of quantification (LOQ) during the initial sampling intervals. This limited release behavior is likely attributed to its high lipophilicity, which favors retention within the internal oil phase of the nanoemulsion droplets and restricts partitioning into the aqueous receptor medium. Similar release limitations have been reported for highly lipophilic compounds in lipid-based delivery systems [7,9].

Trace amounts of DMPBD were first detected only at later time points, specifically after 6 h in PBS containing 1% Tween 80 and after 8 h in PBS:propylene glycol (80:20, *v*/*v*), indicating delayed diffusion from the formulation matrix. The presence of Tween 80 in the receptor medium may have slightly enhanced solubilization and maintained more favorable sink conditions, resulting in earlier detectable release compared with the propylene glycol-containing system [12,21,22,23].

Although a weak linear increase in cumulative release was observed during the later stage of the experiment, suggesting diffusion-limited behavior broadly consistent with the Higuchi concept, the total released amounts remained very low and close to the LOQ. Consequently, reliable kinetic parameters, including steady-state flux (Jss), could not be accurately determined for DMPBD.

#### 2.6.2. Release and Diffusion of Compound D

**Figure 5:**
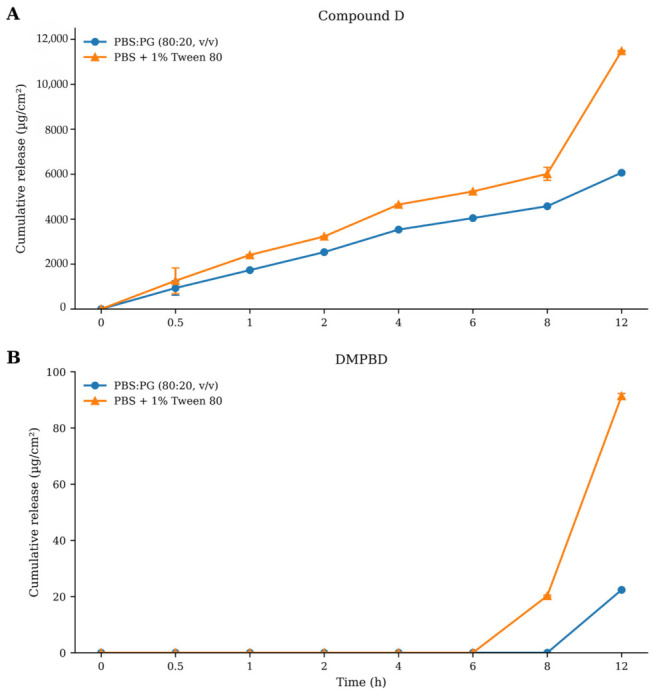
Cumulative release profiles of (**A**) Compound D and (**B**) DMPBD from the nanoemulsion gel over 12 h. Values are mean ± SD (*n* = 3).

In contrast to DMPBD, Compound D exhibited sustained, quantifiable, and substantially greater release throughout the 12 h experimental period in both receptor media, indicating more favorable diffusion behavior from the developed nanoemulsion gel. The cumulative release profile was consistently higher in PBS containing 1% Tween 80 than in PBS:propylene glycol (80:20, *v*/*v*) at all sampling intervals (Figure 5), suggesting that the surfactant present in the receptor phase enhanced solubilization capacity and maintained more effective sink conditions, thereby facilitating continuous diffusion across the membrane [9,15].

**Figure 6:**
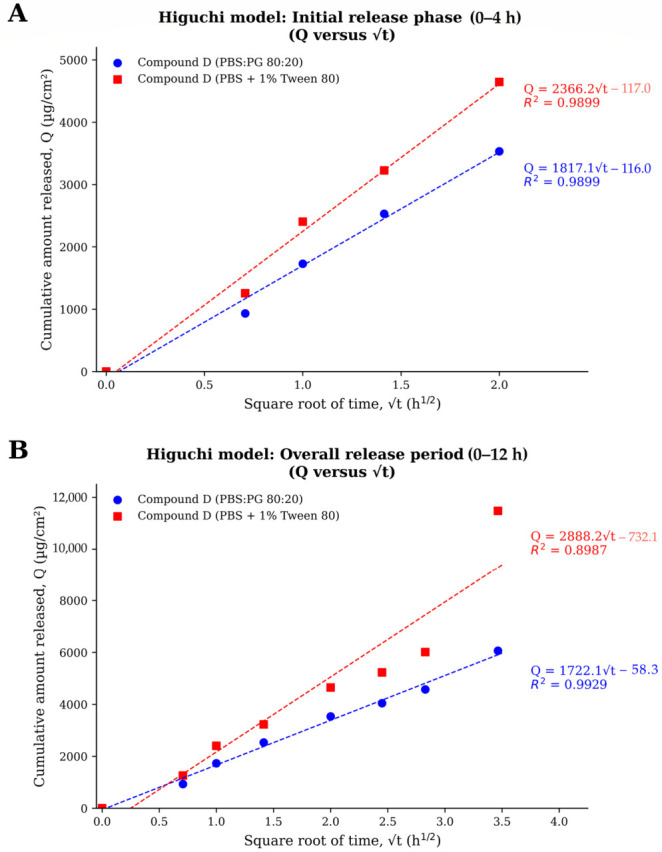
Higuchi model fitting of Compound D release from the nanoemulsion gel. (**A**) Initial release phase (0–4 h), demonstrating rapid release behavior. (**B**) Overall release period (0–12 h), showing cumulative diffusion behavior. Data were fitted using the Higuchi equation (Q versus √t).

The release profile of Compound D demonstrated a biphasic pattern, consisting of an initial rapid release phase during the first 4 h, followed by a slower diffusion-dominated phase over the remaining period. Such behavior is commonly observed in nanoemulsion and matrix-based delivery systems, where compounds located near the droplet interface or loosely associated with the external gel network are released rapidly at early time points, whereas the entrapped fraction diffuses more gradually from the internal oil phase thereafter. To better characterize this behavior, the release data were analyzed separately for the initial phase (0–4 h) and the overall release period (0–12 h) (Figure 6; Table 5 and Table 6).

The cumulative amount of Compound D released at 12 h (Q_12_) was highest in PBS containing 1% Tween 80 (11,478.85 µg/cm^2^), followed by PBS:propylene glycol (80:20, *v*/*v*) (6063.75 µg/cm^2^) (Table 6). During the initial release phase (0–4 h), Higuchi model fitting yielded release constants (k_H_, 0–4 h) of 2366.25 and 1817.13 µg/cm^2^·h^−1/2^ for PBS + 1% Tween 80 and PBS:PG, respectively, with identical correlation coefficients (R^2^ = 0.9899), confirming faster early diffusion in the surfactant-containing receptor medium.

Kinetic model fitting further demonstrated that the release mechanism of Compound D was dependent on receptor medium composition and fitting interval (Table 5). During the overall 0–12 h period, the Higuchi model again provided the best fit in the PBS:propylene glycol system (R^2^ = 0.9929), supporting sustained diffusion-controlled release from the nanoemulsion gel matrix. In contrast, the Korsmeyer–Peppas model showed the highest correlation coefficient in PBS containing 1% Tween 80 (R^2^ = 0.9706), indicating a more complex transport mechanism involving combined diffusion and matrix relaxation effects under enhanced sink conditions. Nevertheless, Higuchi fitting in this medium remained acceptable (R^2^ = 0.8987), suggesting that diffusion still contributed substantially to the overall release process.

The Higuchi release constants obtained from the overall 0–12 h period were 2888.16 µg/cm^2^·h^−1/2^ for PBS + 1% Tween 80 and 1722.10 µg/cm^2^·h^−1/2^ for PBS:PG (Table 6), further confirming the faster diffusion rate in the Tween 80-containing medium. These findings indicate that receptor medium composition strongly influenced the apparent release kinetics of Compound D by altering solubilization efficiency and mass transfer conditions.

Compared with DMPBD, Compound D showed markedly superior release in both receptor media, indicating that molecular physicochemical properties played an important role in determining release behavior from the nanoemulsion system. Compound D likely possesses a more favorable balance between lipophilicity and aqueous affinity, allowing more efficient transfer from the internal oil phase into the receptor medium, whereas the minimal release of DMPBD was likely associated with stronger retention within the lipid core of the formulation.

Overall, these results identify Compound D as the principal releasable marker of the Kamala nanoemulsion gel under the present in vitro conditions. Its favorable release kinetics, higher cumulative release, and stronger diffusion characteristics suggest that Compound D may contribute substantially to the dermal delivery performance of the formulation and may play an important role in the intended anti-inflammatory activity for topical management of knee osteoarthritis.

### 2.7. Translational Implications for Dermal Delivery and Topical Management of Knee Osteoarthritis

The present findings suggest that the developed Kamala nanoemulsion gel has promising potential as a dermal delivery system for supportive management of knee osteoarthritis. The formulation exhibited several desirable characteristics for topical application, including nanoscale droplet size, narrow size distribution, suitable viscosity, and satisfactory physical stability. Collectively, these properties may support uniform application, prolonged residence time on the skin surface, and improved convenience during repeated use.

Nanoemulsion systems offer advantages for topical therapy because their small droplet size provides a large interfacial surface area, which may enhance partitioning of active compounds into the stratum corneum. In addition, surfactant and co-surfactant components may improve interaction with skin lipid domains and facilitate dermal transport through intercellular or appendageal pathways [7,9]. These mechanisms may be beneficial for delivering lipophilic Kamala-derived compounds to tissues surrounding the knee joint.

The superior release of Compound D observed in the receptor medium containing Tween 80 further suggests that maintaining sink conditions and adequate solubilization capacity is important for maximizing release from lipid-based nanoformulations. This observation may provide useful guidance for future formulation optimization aimed at improving the dermal bioavailability of lipophilic phytochemicals [21,22].

However, quantitative skin permeation parameters, such as the apparent diffusion coefficient (Dapp), permeability coefficient (Kp), and steady-state concentration (Qstc), were not evaluated in the present study. Therefore, the actual extent of skin penetration, tissue distribution, and potential accumulation in deeper periarticular tissues cannot yet be confirmed and should be interpreted with caution [10]. Further studies using excised skin or ex vivo permeation models are warranted to verify the delivery potential of the formulation.

Although the formulation demonstrated satisfactory short- to medium-term physical stability under the current study conditions, additional long-term stability monitoring would still be beneficial for future product development and scale-up. Continuous quality assessment and marker-content monitoring using validated analytical methods should also be maintained according to the same quality frameworks applied in the present study [19,20,24].

Overall, these findings provide preliminary evidence that the developed Kamala nanoemulsion gel is a promising modernized dermal delivery platform for Kamala-derived bioactive compounds. With further studies on ex vivo permeation, pharmacokinetic–pharmacodynamic correlation, and in vivo efficacy, the formulation may offer practical potential as a complementary topical option for supportive management of knee osteoarthritis.

### 2.8. Comparative Advantages over the Traditional Kamala Poultice

**Figure 7:**
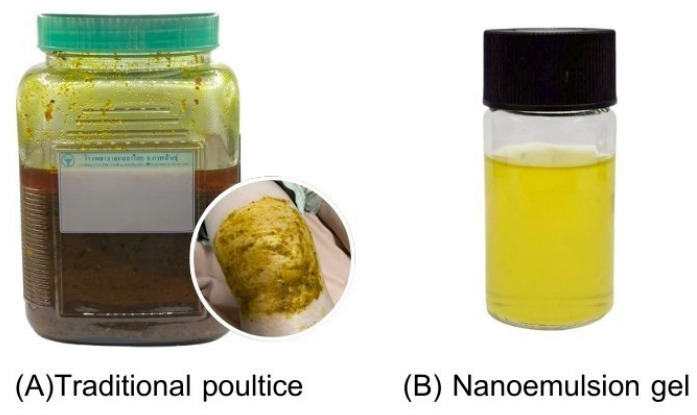
Comparison between traditional poultice (**A**) and nanoemulsion gel (**B**).

Compared with the conventional Kamala poultice, the nanoemulsion gel offers several practical and pharmaceutical advantages, including improved convenience without the need for prolonged wrapping, standardized marker content, enhanced release performance, and improved physicochemical stability [8,10]. These improvements address key limitations of traditional preparation and provide a scientifically validated platform for modern topical application (Figure 7).

Overall, the findings demonstrate that rational nanoformulation can effectively bridge traditional herbal knowledge with contemporary pharmaceutical technology, supporting the further development of Kamala-based products for clinical use [5,6].

## 3. Conclusions

This study successfully established an optimized nanoemulsion gel system for dermal delivery of Kamala-derived phenylbutenoid markers. Coconut oil was identified as the most suitable oil phase, while Tween 80 and n-butanol (2:1) provided optimal interfacial stabilization and phase behavior. Incorporation of the optimized nanoemulsion into an Aristoflex Velvet^®^ gel produced a transparent and physically stable formulation with skin-compatible pH and suitable viscosity.

The developed formulation is in line with recent advances in nanoemulgel-based dermal delivery systems designed to enhance topical bioavailability and patient convenience.

The nanoemulsion gel demonstrated sustained release behavior, with markedly enhanced cumulative release and steady-state flux of Compound D compared with DMPBD. Extended stability testing showed satisfactory short- to medium-term physical stability for up to 60 days under multiple storage conditions.

Although the present work provides a systematic formulation strategy and promising release performance, it should be regarded as an early-stage development study. Further investigations involving ex vivo skin permeation, pharmacokinetic–pharmacodynamic correlation, long-term stability testing, and in vivo efficacy studies are required to confirm the translational potential of the system.

Overall, the developed nanoemulsion gel represents a promising platform for modernizing traditional Thai herbal medicines into standardized topical delivery systems for future clinical application.

## 4. Materials and Methods

### 4.1. Chemicals and Reagents

Coconut oil, sesame oil, palm oil, rice bran oil, and olive oil (analytical grade) were used as oil phases. Tween 80 (polyoxyethylene sorbitan monooleate), Span 80 (sorbitan oleate), and PEG-40 hydrogenated castor oil (Cremophor^®^ RH40) were used as surfactants. n-butanol, absolute ethanol, isopropyl alcohol, and propylene glycol were employed as co-surfactants. Unless otherwise specified, all chemicals were of analytical grade and used as received without further purification.

Gelling agents used in this study included Aristoflex^®^ Velvet, Aristoflex^®^ AVC, Sepimax™ ZEN, and xanthan gum. HPLC-grade methanol, acetonitrile, and water were used for chromatographic analysis. Synthetic cellulose acetate membranes (0.45 µm pore size) for diffusion studies were obtained from Sartorius (Göttingen, Germany). Phosphate-buffered saline (PBS, pH 7.4) and Tween 80^®^ aqueous solutions were freshly prepared in-house according to standard laboratory protocols [10,15].

The phenylbutenoid reference standards, (*E*)-1-(3,4-dimethoxyphenyl)butadiene (DMPBD) was obtained from LGC Standards (Bury, UK). The compound (*E*)-4-(3′,4′-dimethoxyphenyl)but-3-en-1-ol (Compound D) was purchased from Chengdu Biopurify Phytochemicals Ltd. (Chengdu, China).

### 4.2. Plant Extraction

All medicinal plants were harvested in November 2024 and characterized by Mr. Boonyong Buabuppha, a recognized local expert in traditional herbal medicine and Methajarn (master scholar) of the University of Life, Kalasin Province, Thailand, based on ethnobotanical knowledge. Kamala remedy, a standardized extract containing phenylbutenoid markers, was macerated with 3 L 95% ethanol, occasionally shaken for 7 days and then filtered with Whatman paper No. 1. Finally, the Kamala extract was collected and kept in air-tight container protected from light for further solubilization of phenylbutenoids in oils—sesame, palm, rice bran, coconut, and olive oils [25].

### 4.3. GC–MS Analysis of Phenylbutenoid Markers for Oil Screening

Three milliliters of Kamala extract was added in 6 mL of each oil and placed into a shaking incubator with programmed at 40 °C, 150 rpm for 48 h (JS Research Inc., Gongju, Republic of Korea). The resulting oil solutions were filtered through a PTFE 0.45 µm membrane and 1 µL was injected in splitless mode. The solubility of (*E*)-1-(3,4-dimethoxyphenyl)butadiene (DMPBD) and (*E*)-4-(3′,4′-dimethoxyphenyl)but-3-en-1-ol (Compound D) in different oils was evaluated using gas chromatography (Agilent 7890B)–mass spectrometry (Agilent 5977B) equipped with an electron ionization (EI) source operating at 70 eV. The injector temperature was set at 270 °C, and the helium carrier gas was set at a constant flow rate of 1 mL/min. Chromatographic separation was performed on a DB-5MS capillary column (30 m × 0.25 mm, 0.25 µm film thickness, Agilent Technology, Santa Clara, CA, USA). The oven temperature was programmed from 50 °C (held for 3 min) to 300 °C at a rate of 10 °C/min and maintained for 12 min. Compound identification was achieved by comparison with the NIST 17 mass spectral library using a similarity threshold greater than 85% [26].

### 4.4. Construction of Pseudo-Ternary Phase Diagrams

Pseudo-ternary phase diagrams were constructed using the water titration method. Surfactant–co-surfactant mixtures (Smix) composed of Tween 80 and n-butanol were prepared at weight ratios of 3:1, 2:1, 1:1, 1:2, and 1:3. Each Smix ratio was subsequently mixed with coconut oil at oil-to-Smix mass ratios ranging from 1:9 to 9:1. Distilled water was gradually added under continuous magnetic stirring at 600 rpm until the onset of turbidity or phase separation was visually observed [27].

The phase behavior of each system was mapped onto triangular coordinates using an online ternary plotting tool (https://ternaryplot.com/; accessed on 30 September 2025). Clear, isotropic systems exhibiting low viscosity and no observable phase separation were classified as nanoemulsion regions [28].

### 4.5. Preparation of Nanoemulsions by Ultrasonication

Based on the phase diagram analysis, three formulations—K1 (40% Smix), K2 (50% Smix), and K3 (60% Smix)—were selected. Appropriate amounts of Kamala extract, oil phase, and Smix were mixed and pre-emulsified using a vortex mixer for 5 min. Ultrasonication was performed using a VCX 750 probe sonicator (Sonics & Materials Inc., Newtown, CT, USA) at 40% amplitude in pulse mode (30 s on/10 s off) for a total processing time of 5 min. To prevent thermal degradation, the samples were maintained in an ice bath, following established nanoemulsion preparation protocols [10,12]. ensuring that the temperature remained below 35 °C throughout the process.

Droplet size and polydispersity index (PDI) were measured immediately after preparation and re-evaluated after 24, 48, and 72 h [18,21].

### 4.6. Particle Size, PDI, and Stability Studies

Particle size and polydispersity index were measured by dynamic light scattering (DLS) using a Zetasizer Nano ZS (Malvern Instruments, Worcestershire, UK). Samples were diluted 1:100 with filtered distilled water to reduce multiple scattering effects during analysis [12,28]. All measurements were performed in triplicate.

Physical stability was evaluated by centrifugation at 5000 rpm for 30 min, thermal cycling between 4 °C and 40 °C (24 h at each temperature for three cycles), and visual inspection for clarity, phase separation, and creaming [10,27].

### 4.7. Preparation of Nanoemulsion Gels

Nanoemulsion gels were prepared by dispersing each gelling agent (0.5% and 1% *w*/*w*) in distilled water and allowing hydration for 3 h. The optimized nanoemulsion formulation (K2) was then incorporated under gentle mechanical stirring at 300 rpm to minimize air entrapment. The resulting gels were equilibrated at 25 °C for 24 h prior to further evaluation [8,13].

### 4.8. Physicochemical Evaluation of Gels

#### 4.8.1. pH Measurement

The pH of the gels was measured at 25 °C using a calibrated digital pH meter SevenCompact model (Mettler Toledo, Greifensee, Switzerland) after dilution with distilled water (1:10, *w*/*v*), following previously reported procedures for semisolid topical formulations [21,23,29].

#### 4.8.2. Viscosity

Viscosity measurements were performed using a Brookfield DV1 viscometer equipped with an LV spindle set (AMETEK Brookfield, Middleboro, MA, USA). Measurements were conducted on days 1, 7, 14, 30, and 60 at storage temperatures of 5, 25, and 40 °C [13,23].

#### 4.8.3. Visual Appearance

Gels were visually examined for homogeneity, transparency, syneresis, and color changes throughout the study period [19,30,31].

### 4.9. In Vitro Release Study Using Franz Diffusion Cells

In vitro permeation studies were performed using vertical Franz diffusion cells (PermeGear, Hellertown, PA, USA); diffusion area ~1.77 cm^2^, receptor volume ~16 mL, water-jacketed) following established procedures [9,15,32,33]. Synthetic cellulose acetate membranes were used to assess comparative release behavior and formulation-dependent diffusion under controlled conditions.

Two receptor media were employed: (i) PBS (pH 7.4) containing 1% Tween 80 and (ii) PBS (pH 7.4): propylene glycol (80:20, *v*/*v*). Quantification of DMPBD and Compound D was performed by HPLC, and flux and release kinetics were evaluated using the Higuchi model for diffusion-controlled release and the Korsmeyer–Peppas model for anomalous transport behavior involving diffusion and matrix relaxation mechanisms [15,22].

Chromatographic analysis of Phenylbutenoids was conducted using a high-performance liquid chromatography system, Agilent 1260 Infinity II model (Agilent Technologies, Santa Clara, CA, USA). Chromatographic system was performed on a ZORBAX^®^ Eclipse Plus C_18_ column (4.6 × 250 mm, 5 μm particle size) under 25 °C controlled temperature. The mobile phase consisted of 0.1% trifluoroacetic acid (solvent A) and acetonitrile (solvent B) programmed with gradient elution: 0–5 min, 10–20% B; 5–10 min, 20–40%B; 10–20 min, 40–60% B; 20–25 min, 60–90% B; 25–30 min, 90–100% B; 30–40 min, 100–10% B; and 40–45 min, 10% B with flow rate at 0.8 mL/min. The injection volume was set at 10 μL. Photodiode array detection was conducted at specific wavelengths to each compound: 254 nm for Compound D and 284 nm for DMPBD. The retention times of Compound D and DMPBD were approximately 14.2 min and 26.0 min, respectively. Identification of Compound D and DMPBD in samples were confirmed by retention times and UV spectra of the corresponding standard solution.

The validated HPLC method ensured reliable quantification of phenylbutenoid markers in release studies, supporting the accuracy of the diffusion and kinetic analysis. The HPLC analytical method was validated in accordance with ICH Q2(R1) guidelines. Linearity was confirmed over the tested concentration ranges, with correlation coefficients (R^2^) of 0.9997 for Compound D and 1.000 for DMPBD, indicating excellent linear relationships between peak area and concentration [24].

The limit of detection (LOD) and limit of quantification (LOQ) were calculated based on the standard deviation of the response and the slope of the calibration curve. The LOD and LOQ values were 0.68 and 2.07 µg/mL for Compound D, and 0.82 and 2.50 µg/mL for DMPBD, respectively.

Accuracy was evaluated at two concentration levels (50 and 100 µg/mL), showing recovery values within 98–102%, indicating good agreement with accepted criteria. Precision assessment demonstrated excellent repeatability, with %RSD values below 1% for both analytes [21,24].

### 4.10. Statistical Analysis

All experiments were conducted in triplicate, and results are expressed as mean ± standard deviation (SD). Statistical analysis was performed using the IBM SPSS Statistics software version 29 (IBM Corp., Armonk, NY, USA). One-way analysis of variance (ANOVA) followed by Tukey’s post hoc test was used to assess statistical significance at *p* < 0.05.

## Figures and Tables

**Table 1:** Solubilization efficiency of DMPBD and Compound D in oils (mean ± SD).

Oil	DMPBD (GC–MS Peak Area)	Compound D (GC–MS Peak Area)
Sesame Oil	733,291.0 ± 163,163.9 ^d^	58,568.3 ± 44,522.3 ^e^
Palm Oil	7,095,506.3 ± 132,621.7 ^b^	3,939,167.0 ± 127,801.3 ^b^
Rice Bran Oil	6,210,990.7 ± 171,707.0 ^c^	3,149,921.0 ± 109,385.3 ^c^
Coconut Oil	7,726,887.0 ± 82,326.5 ^a^	4,827,591.3 ± 136,944.0 ^a^
Olive Oil	5,930,135.3 ± 85,295.5 ^c^	1,980,225.0 ± 164,589.2 ^d^

Values are expressed as mean ± standard deviation (SD) of triplicate determinations (*n* = 3). Solubilization efficiency was assessed based on GC–MS chromatographic peak areas of DMPBD and Compound D dissolved in each oil phase. Different letters indicate significant differences in the same column (*p* < 0.05).

**Table 2:** Selection of surfactants for nanoemulsion formulation based on coconut oil solubilization capacity.

Surfactant (15%*w*/*w*)	Maximum Coconut Oil Incorporated (µL)
Tween 80 (Polysorbate 80)	4 ± 0.00 ^a^
Span 80 (Sorbitan Oleate)	2 ± 0.00 ^b^
PEG-40 Hydrogenated Castor Oil	4 ± 0.00 ^c^

Values are expressed as mean ± SD (*n* = 3). Different superscript letters indicate statistically significant differences (*p* < 0.05; Kruskal–Wallis test followed by Dunn’s multiple comparison test).

**Table 3:** Physicochemical characteristics of nanoemulsion formulations containing different Smix concentrations.

Formulation (Smix, %)	Particle Size (nm) After Ultrasonication	PDI After Ultrasonication	Particle Size (nm) After 72 h	PDI After 72 h	*p*-Value ^†^ (Size)	*p*-Value ^†^ (PDI)
K1 (40%)	68.30 ± 18.50 ^b^	0.235 ± 0.033 ^a^	223.63 ± 56.20 ^a^	0.507 ± 0.074 ^a^	0.010	0.004
K2 (50%)	77.92 ± 8.34 ^b^	0.199 ± 0.043 ^a^	130.89 ± 29.16 ^b^	0.195 ± 0.013 ^c^	0.039	0.885
K3 (60%)	193.60 ± 55.97 ^a^	0.271 ± 0.045 ^a^	164.70 ± 53.59 ^ab^	0.319 ± 0.138 ^b^	0.553	0.597

Values are expressed as mean ± SD (*n* = 3). Different superscript letters (^a–c^) within the same column indicate statistically significant differences among formulations (*p* < 0.05, one-way ANOVA followed by Tukey’s post hoc test). ^†^ *p*-values represent comparisons between measurements taken immediately after ultrasonication and after 72 h within the same formulation using paired *t*-test. Particle size and polydispersity index (PDI) were determined by dynamic light scattering (DLS).

**Table 4:** Viscosity stability of the Aristoflex Velvet 1% nanoemulsion gel during storage under different temperature conditions.

Storage Condition	Day 1 Viscosity (cP)	Day 60 Viscosity (cP)	% Change	*p*-Value ^†^
5 °C	3804.00 ± 31.18	4272.00 ± 0.00	+12.30%	<0.001
25 °C	3804.00 ± 31.18	3680.00 ± 95.00	−3.26%	0.421
40 °C	3804.00 ± 31.18	3966.00 ± 0.00	+4.26%	0.018

Viscosity was measured using a Brookfield DV1 viscometer equipped with spindle LV-64 at 25 ± 1 °C, with a measurement time of 60 s per step. Values are expressed as mean ± SD (*n* = 3). ^†^ *p*-values represent comparison between Day 1 baseline and Day 60 values at each storage temperature using paired *t*-test. A value of *p* < 0.05 was considered statistically significant.

**Table 5:** Comparison of kinetic model fitting for Compound D release from the nanoemulsion gel during the initial (0–4 h) and overall (0–12 h) release phases.

Model	PBS:PG (0–4 h), R^2^	PBS + 1% Tween 80 (0–4 h), R^2^	PBS:PG (0–12 h), R^2^	PBS + 1% Tween 80 (0–12 h), R^2^
Zero-order	0.9554	0.9792	0.9131	0.9439
First-order	0.9687	0.9875	0.9741	0.9446
Higuchi	0.9899	0.9899	0.9929	0.8987
Hixson-Crowell	0.9518	0.9826	0.8955	0.7636
Korsmeyer-Peppas	0.9724	0.9913	0.9222	0.9706

The highest R^2^ value in each column indicates the best-fitting kinetic model for the corresponding receptor medium and release interval. R^2^ = coefficient of determination; PBS:PG = phosphate-buffered saline containing propylene glycol (80:20, *v*/*v*).

**Table 6:** Comparison of Compound D release parameters during the initial phase (0–4 h) and overall release period (0–12 h).

Medium	Initial Release Phase (0–4 h)		Overall Period (0–12 h, Higuchi Model)		Q_12_ (µg/cm^2^)
	K_H_ (µg/cm^2^·h^−1/2^)	R^2^	K_H_ (µg/cm^2^·h^−1/2^)	R^2^	Q_12_ (µg/cm^2^)
PBS:PG (80:20, *v*/*v*)	1817.13	0.9899	1722.10	0.9929	6063.75
PBS + 1% Tween 80	2366.25	0.9899	2888.16	0.8987	11,478.85

Abbreviations: kH = Higuchi release constant obtained from linear regression of cumulative amount released (Q) versus square root of time (√t); kH (0–4 h) = release constant during the initial phase; kH (0–12 h) = release constant over the overall release period; R^2^ = coefficient of determination; Q_12_ = cumulative amount released at 12 h.

## Data Availability

Data will be available on reasonable request from the corresponding author.

## Abbreviations

The following abbreviations are used in this manuscript:

Compound D	(*E*)-4-(3′,4′-dimethoxyphenyl)but-3-en-1-ol
DMPBD	(*E*)-1-(3,4-Dimethoxyphenyl)butadiene
GC–MS	Gas Chromatography–Mass Spectrometry
DLS	Dynamic Light Scattering
PDI	Polydispersity Index
PBS	Phosphate-Buffered Saline
Smix	Surfactant–Co-surfactant Mixture
